# *OCA2* common variant NM_000275.3:c.574-19A>G affects splicing and is pathogenic.

**DOI:** 10.1016/j.ymgmr.2026.101331

**Published:** 2026-06-17

**Authors:** Modibo Diallo, Alicia Defay-Stinat, Claudio Plaisant, Sabine Derrien, Elina Mercier, Sophie Javerzat, Shahram Mesdaghi, Daniel J. Rigden, Eulalie Lasseaux, Vincent Michaud, Benoit Arveiler

**Affiliations:** a Laboratoire Maladies Rares, Génétique et Métabolisme, Bordeaux University INSERM U1211, Bordeaux, France; b Service de Génétique Médicale, Centre Hospitalier Universitaire de Bordeaux, Bordeaux, France; c Electrophysiology Department, Rothschild Foundation Hospital, Paris, France; d Institute of Systems, Molecular and Integrative Biology, University of Liverpool, Liverpool, United Kingdom; e Computational Biology Facility, MerseyBio, University of Liverpool, Crown Street, Liverpool, United Kingdom

**Keywords:** OCA, Intronic variant, NGS, Splicing, Common variant

## Abstract

Albinism is characterized by generalized hypopigmentation and ocular features resulting from impaired melanin biosynthesis. Most known pathogenic variants are rare (MAF < 0.001) and found in coding regions. The role of non-coding variants, especially those with higher allele frequencies, is generally not investigated. Our next generation sequencing panel that includes the entire sequence of five major albinism genes (*TYR*, *OCA2*, *SLC45A2*, *GPR143* and *HPS1*) identified compound *OCA2* heterozygosity in a patient with a rare (MAF:0,0003) coding variant, NM_000275.3:c.1025 A > G;p.(Tyr342Cys) and a more common intronic variant, NM_000275.3:c.574-19 A > G (MAF:0,0087, with 80 homozygotes in the control population GnomADv4.1.0). *In silico* prediction tools indicated that this intronic variant could alter splicing. RT-PCR analysis on RNA extracted from the patient's blood revealed the skipping of exons 6 and 7, which resulted in the in-frame deletion of 78 amino acids. Protein modelling suggested that this deletion disrupts the GOLD-like domain and leads to the loss of conserved N-glycosylation sites, likely impairing protein folding and intracellular trafficking. These findings provided strong evidence for a deleterious effect on OCA2 function. Therefore, the variant was classified as likely pathogenic, allowing to establish the diagnosis in the patient. The relatively high allele frequency of this variant suggests that it behaves as a hypomorphic allele leading to disease in a compound heterozygous state. This underscores that intronic variants outside the canonical splice sites must be taken in consideration and functionally tested, and that common variants should not be systematically discarded in the diagnosis of albinism, and, similarly, of other rare diseases.

## 1 Introduction

Albinism is a group of rare genetic diseases characterized by marked phenotypic and genetic heterogeneity. It results from defects in melanin biosynthesis and is associated with a variable degree of generalized hypopigmentation, and ocular anomalies including nystagmus, retinal hypopigmentation, photophobia, foveal hypoplasia, misrouting at the optic chiasma, and reduced visual acuity. To date, pathogenic variants involved in albinism have been identified in 21 genes, reflecting the substantial genetic complexity of the disease [1], [2]. Three main clinical forms are described: Oculocutaneous albinism (OCA) with eight subtypes (OCA1 to 8), X-linked ocular albinism (OA1) and syndromic forms including Hermansky-Pudlak Syndrome (HPS) and Chediak-Higashi Syndrome (CHS). The prevalence of the disease greatly differs worldwide, varying from ∼1/4000 in Africa to ∼1/12000 in Europe [3].

The molecular diagnosis of rare genetic disorders most commonly relies upon the search for rare variants (with an allelic frequency < 0.1% in the general population) in the coding exons and splice consensus sequences. It has however been shown that variants located in the introns and in flanking sequences may have disease-causing effects by altering either RNA splicing, leading to skipping of one or more exons or the inclusion of pseudoexons [4], [5], or expression regulation when located in promoters or enhancer elements [6], [7].

A ∼ 70% diagnostic rate can be achieved in patients with albinism by sequencing the exons and exon-intron junctions of the 21 albinism genes, completed by a search for copy number variations [8]. We recently improved our next generation sequencing diagnostic panel by including the entire sequence (exons, introns, flanking sequences) of 5 major genes, namely *TYR*, *OCA2*, *SLC45A2*, *GPR143* and *HPS1* [9].

We present here a patient in whom the search for rare variants (MAF ≤ 0.001; minor allele frequency) initially identified a single pathogenic variant in the *OCA2* gene. We thereafter extended the search to more common variants (0.001 ≤ MAF ≤ 0.05) and identified a relatively common intronic variant, NM_000275.3:c.574-19 A > G (MAF:0,0087), which affects splicing of the *OCA2* RNA.

## 2 Methods

### 2.1 Patient

The patients were referred for molecular diagnosis of albinism. Informed consent for genetic analysis was collected for the patients and their parents. This study was approved by the local authority (Comité de Protection des Personnes Sud Ouest et Outre Mer III).

### 2.2 Albinism NGS panel sequencing

The 21 known albinism genes, including that for FHONDA were analyzed (*TYR*, *OCA2*, *TYRP1*, *SLC45A2*, *SLC24A5*, *LRMDA*, *DCT*, *GPR143*, *HPS1–11*, *LYST*, *SLC38A8*). This included for all genes the exons and intron-exon junctions. The introns and flanking sequences were also analyzed for *TYR* (OCA1), *OCA2* (OCA2), *SLC45A2* (OCA4), *GPR143* (OA1), and *HPS1* (HPS1). Coordinates of all sequences included were defined by comparison with the reference sequence GRCh38 and are available upon request. Next generation sequencing and bioinformatic analysis of the data were performed as described in [9].

### 2.3 RT-PCR analysis

Total RNA was isolated from white blood cells, using the PAXgene Blood RNA kit (Qiagen), as indicated in the manufacturer's protocol. One μg of total RNA from each sample was reverse-transcribed into cDNA using a cDNA synthesis Kit (Thermo Fisher Scientific). Reverse Transcription-PCR primers were designed (primer3 version 4.1.0; https://primer3.ut.ee/) based on the MANE transcript of the *OCA2* mRNA NM_000275.3. Primers for RT-PCR were 5’CAGATGTCCAGCTCCAGGTC3’ in exon 3 (forward) and 5’TGATGGACACCGTCTCTCTG3’ (reverse) located at the exon 8-exon 9 junction. Touchdown PCR was performed for 10 cycles with Tm ranging from 71° to 61 °C for 10 cycles, followed by 30 cycles (95° for 30s, 61° for 15 s, and 72° for 20s). PCR products were separated by 2% agarose gel electrophoresis and Sanger sequenced (Eurofins).

### 2.4 Sanger sequencing

Sanger sequencing was carried out by Eurofins genomics (Ebersberg, Germany) using the cycle sequencing technology (dideoxy chain termination/cycle sequencing) on an ABI 3730XL (Applied Biosystems) sequencing machine. This technique was used to confirm presence of the *OCA2* intronic variant, to implement segregation analysis in the parents, and to characterize RT-PCR products.

### 2.5 Protein modelling

Protein model building was performed using ColabFold v1.1.5 [10] implementation of AlphaFold2 [11] on an Ubuntu 20.04.6 workstation AMD Ryzen Threadripper 2990WX 32 Core CPU (3.0 GHz) with 64GB RAM. GPU acceleration was performed by an ASUS TUF GeForce RTX 3080 OC LHR 12GB GDDR6X Ray-Tracing Graphics Card, 8960 Core, 1815 MHz Boost. The ColabFold ‘template’ search was enabled along with the energy minimization refinement flag (‘amber’) and the GPU relax feature (−-use-gpu-relax). The OPM server [12] was used to position the OCA2 models into the lipid bilayer.

## 3 Results and discussion

### 3.1 Phenotypical description

The patient was a 25-year-old French woman presenting with congenital nystagmus since birth, photophobia with brown irises, and low visual acuity. OCT examination revealed foveal hypoplasia (fovea plana) in both eyes. No transillumination of the iris was observed upon slit lamp examination. She had pink skin, light brown hair and brown eyes, quite similar to her parents who both had pink skin, brown hair and brown eyes. In total, the patient was diagnosed with moderate oculocutaneous albinism (OCA) according to clinical findings.

### 3.2 Molecular analysis

**Fig. 1:**
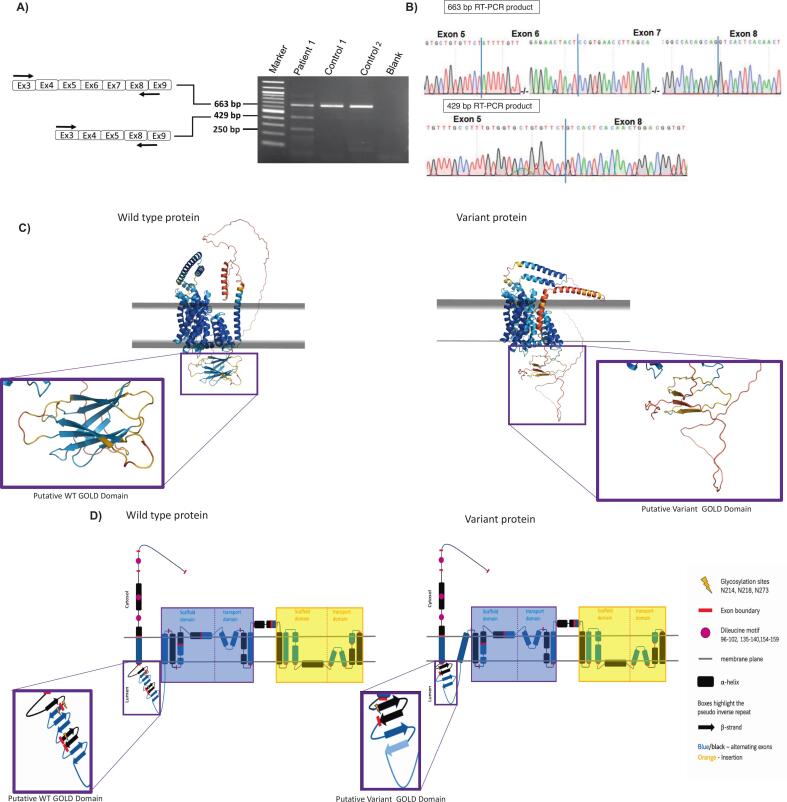
Characterization of variant NM_000275.3:c.574-19 A > G. A) RT-PCR using primers derived in exon 3 and at the junction between exons 8 and 9. A schematic representation of the 429 and 633 bp RT-PCR fragments is provided on the left; black arrows represent the primers used. Gel electrophoresis of RT-PCR products is shown on the right for the patient and 2 control individuals without a known genetic disease. Sizes of RT-PCR products are indicated on the left. B) Sanger sequences of the 429 and 633 bp RT-PCR products show the nucleotides at the junctions between exons, represented by vertical blue bars. −/− represents the middle of the exons, the sequence of which is not shown. C) AlphaFold2 generated OCA2 models. The wild type protein is shown on the left and the variant-bearing protein on the right. Structures are colored by pLDDT (predicted Local Distance Difference Test) scores, ranging from blue (high confidence) to red (low confidence). The pLDDT score is an indicator of the confidence in the predicted protein structure, with values ranging from 0 to 100. Higher scores denote higher confidence in the accuracy of the predicted structure for specific protein regions. Grey planes indicate membrane boundaries as predicted by the OPM server [12]. D) OCA2 topology: The wild-type protein is presented on the left and the variant-bearing protein on the right. The wild-type topology is adapted from [18], with minor revisions based on new data. The deletion removes the amino acid sequence corresponding to the first four β-strands of the predicted GOLD domain, while retaining the sequence encoding the second set of four β-strands. Structural modelling, however, predicts preservation of only the first three β-strands within the second set, with the fourth strand partially disrupted and replaced by an unstructured region (shown in pale blue).

Next generation sequencing of the patient's genomic DNA identified a heterozygous rare variant, NM_000275.3:c.1025 A > G;p.(Tyr342Cys) in the *OCA2* gene, which was inherited from her father (data not shown) and could be classified as likely pathogenic according to the American College of Medical Genetics (ACMG) criteria (PM2 PM3 PP3 PP5) [13]. No other rare variant (MAF <0.001) was found in the *OCA2* gene nor in the other 20 albinism genes. Extending our search to more common variants identified variant NM_000275.3:c.574-19 A > G;p? in the heterozygous state. This variant located in intron 5 of *OCA2*, has a MAF of 0.0087, with 13,844 heterozygous and 80 homozygous individuals in the control population GnomADv4.1.0. It was inherited from the mother (data not shown) and was predicted to affect splicing by various *in silico* prediction tools. SPiP [14] predicted a complex alteration of the splicing process with 100% probability, RNA Splicer (https://rddc.tsinghua-gd.org/ai/rna-splicer) predicted skipping of exon 6, and Max Ent Scan (through the Alamut Visual Plus aggregator, Sophia Genetics) predicted the creation of a novel cryptic acceptor site at position chr15:g.28022592 T > C causing the inclusion of 18 bp from intron 5. Splice AI [15] scores were not significant [acceptor gain: 0.02 (−1); acceptor loss: 0.08 (−19)]. RT-PCR analysis was performed using primers designed in exon 3 and at the junction between exons 8 and 9 (Fig. 1A) on RNA extracted from blood sampled in a PAXgene tube (Qiagen), as previously described [9], [16]. Three RT-PCR products were observed for the patient and were Sanger sequenced (Fig. 1A and B). The 663 bp product, also found in the 2 controls (individuals without a known genetic disease), corresponded to the wild-type cDNA. The 429 bp product, only seen in the patient, demonstrated skipping of exons 6 and 7. The ∼250 bp band, absent from the controls, did not correspond to *OCA2*-derived sequences but to ribosomal RNA 28S and is therefore aspecific.

Alternative splicing involving exons 6 and 7 has not been described in the literature to our knowledge. GTeX (Genotype Tissue Expression Project, https://gtexportal.org/home/gene/OCA2#gene-transcript-browser-block) describes a short *OCA2* isoform, ENST00000445578.5, that contains exons 1, 2, 3, 4, 5, and 8 only. In the RT-PCR assay used here, one primer was in exon 3 and the other one at the junction between exons 8 and 9. Therefore the 429 bp RT-PCR product we amplified in the patient cannot correspond to isoform ENST00000445578.5 but more likely corresponds to an exon 6–7 skipping of the full-length mRNA isoform NM_000275.3.

Noticeably, NM_000275.3:c.574-19 A > G;p? was previously described in a Tanzanian patient with albinism in *trans* to the common *OCA2* exon 7 deletion [17]. We also found this variant in 3 patients of our cohort of patients (cohort previously described in [9]), in whom another loss of function variant was identified. For these patients the phase was not determined because segregation analysis could not be performed or was not informative, and RT-PCR analysis was not performed since appropriate samples were not available.

In addition, NM_000275.3:c.574-19 A > G was identified in a GWAS for blue eyes (referred as rs145242923 in [18]), as were other common *OCA2* variants: rs12913832 in intron 86 of *HERC2* [19], [20], NM_000275.3:c.1256G > A;p.Arg419Gln [21], [22], [23] and NM_000275.3:c.1065G > A;p.Ala355= [21]. Interestingly, NM_000275.3:c.1065G > A was recently shown to increase the skipping of *OCA2* exon 10 [24]. These observations further support the pathogenicity of NM_000275.3:c.574-19 A > G. Although it only seems to be correlated to dilute eye colour in the heterozygous or homozygous state, it is likely pathogenic if found compound heterozygous with a LOF variant in *trans*.

### 3.3 Protein modelling

Skipping of exons 6 and 7 is predicted to cause an in-frame deletion of 78 amino acids in the OCA2 protein. ColabFold [10] was employed to construct models for visualizing the structural consequences of the 78-amino acid deletion. The model with the highest confidence score was chosen for detailed analysis. AlphaFold2 modelling [11] of the wild type OCA2 protein predicts that the initial luminal 130-residue loop forms an eight-stranded beta sandwich, which exhibits strong structural similarities to a GOLD (Golgi Dynamics) domain [25]. The Human OCA2 protein contains three evolutionarily conserved consensus N-glycosylation sites (Asn 214, 218, and 273) within this putative GOLD domain [26], which may be crucial for its trafficking between the ER and Golgi apparatus before localization at melanosomal membranes [27].

The exon 6–7 skip variant results in the loss of four beta sheets from the putative GOLD-like domain, consequently eliminating the conserved Asn214 and Asn218 glycosylation sites. If the GOLD-like domain is indeed responsible for OCA2 trafficking, the exon 6–7 skip would impair or even halt OCA2 transport from the ER to its final destination within the melanosomal membrane, potentially leading to total inhibition of OCA2 melanosome substrate transport.

Additionally, the helical packing of the N-terminal scaffold domain of OCA2 is disrupted, as indicated by lower predicted Local Distance Difference Test (pLDDT) scores in this region compared to the wild-type protein (see Fig. 1C for the pLDDT-mapped model). Alterations in the protein sequence due to exon deletion can generate regions of the protein structure that lack or conflict with the evolutionary covariance signal utilized by AlphaFold2 for modelling. Consequently, these regions may not be accurately captured by the predictive model, resulting in reduced confidence scores. The regions with lower pLDDT scores suggest diminished certainty about the precise 3D structure of the protein in those areas. The deleted sequences might introduce structural variability or disrupt existing structural motifs, leading to the displacement of the first N-terminal alpha helix (see Fig. 1D for a schematic comparison between the wild-type and variant proteins).

Our model reveals that, providing the protein is produced, exon 6–7 skipping severely compromises the structural integrity of the GOLD-like domain of OCA2 and provides strong evidence for a deleterious effect on OCA2 function.

Hence variant NM_000275.3:c.574-19 A > G;p.(Ile192_Gln269del) can be classified as likely pathogenic (PM3 PM4 PS3 BS2) [13].

## 4 Conclusion

The “rare disease - rare variant” paradigm has long been regarded as a rule in the search for causal variants of rare genetic diseases. More recently, the role of common variants has been highlighted, particularly in the context of neurodevelopmental disorders [28], [29], [30]. For oculocutaneous albinism type 1 as well, we have shown that common variants of the *TYR* gene contribute to pathogenicity [31].

In the present study, we identified variant NM_000275.3:c.574-19 A > G;p? in the *OCA2* gene, with a MAF of 0.0087, for which there are 80 homozygotes in GnomADv4.1.0. The presence of homozygotes in the control population suggests that this variant is hypomorphic and is pathogenic only when present in the compound heterozygous state with a loss-of-function variant as is the case in the patient presented here.

We showed that the variant causes the skipping of exons 6 and 7 leading, providing the protein is synthetized, to disruption of the OCA2 protein structure and is therefore pathogenic.

This further supports previous data indicating the involvement of common variants in albinism [24], [31]. We therefore recommend that whole gene sequencing (*i.e.* including the introns) is performed by clinical laboratories, and that the occurrence of common variants is thoroughly examined if only one rare variant is identified. More generally, this may apply to other rare genetic diseases.

## CRediT authorship contribution statement

**Modibo Diallo:** Writing – review & editing, Writing – original draft, Visualization, Validation, Resources, Methodology, Investigation, Formal analysis, Data curation, Conceptualization. **Alicia Defay-Stinat:** Methodology, Investigation, Formal analysis. **Claudio Plaisant:** Methodology, Investigation. **Sabine Derrien:** Resources. **Elina Mercier:** Methodology. **Sophie Javerzat:** Writing – review & editing. **Shahram Mesdaghi:** Writing – review & editing, Visualization, Validation, Methodology, Investigation. **Daniel J. Rigden:** Writing – review & editing, Visualization, Validation, Methodology, Investigation. **Eulalie Lasseaux:** Resources. **Vincent Michaud:** Writing – review & editing, Validation, Resources, Funding acquisition, Formal analysis. **Benoit Arveiler:** Writing – review & editing, Writing – original draft, Visualization, Validation, Supervision, Resources, Project administration, Investigation, Funding acquisition, Formal analysis, Data curation, Conceptualization.

## Funding

10.13039/501100001665Agence Nationale de la Recherche (France) (grant number ANR-21-CE17-0041-01).

Genespoir, French Albinism Association, 2024 grant.

Conseil Régional Nouvelle Aquitaine (France) (convention 2018-1R30113–8473520).

Bordeaux University Hospital (France) (CHUBX 2021/40; ClinicalTrials.gov ID NCT05696912).

## Declaration of competing interest

The authors declare no conflict of interest.

## Data Availability

Data will be made available on request.
